# Immune Mechanisms and Biomarkers in Systemic Lupus Erythematosus

**DOI:** 10.3390/ijms25189965

**Published:** 2024-09-15

**Authors:** Ioannis Parodis, Christopher Sjöwall

**Affiliations:** 1 Division of Rheumatology, Department of Medicine Solna, Karolinska Institutet and Karolinska University Hospital, SE-171 77 Stockholm, Sweden; 2 Department of Rheumatology, Faculty of Medicine and Health, Örebro University, SE-701 82 Örebro, Sweden; 3 Division of Inflammation and Infection/Rheumatology, Department of Biomedical and Clinical Sciences, Linköping University, SE-581 85 Linköping, Sweden; christopher.sjowall@liu.se

The immense heterogeneity of the chronic, inflammatory, autoimmune disease systemic lupus erythematosus (SLE), both with regard to immunological aberrancies and clinical manifestations, poses diagnostic difficulties and challenges in the management of patients [1,2,3]. This is underlined by the lack of generally accepted diagnostic criteria and the numerous clinical trial failures. However, the treatment landscape has witnessed substantial changes during the last decade, facilitated by advances in biotechnology and hence new knowledge on the pathophysiology of SLE.

SLE predominantly affects women, with the onset of the disease typically occurring during their reproductive years. Early diagnosis and treatment initiation are important for the prevention of organ damage accrual [4]. The chronic nature of the disease and its varying course necessitate regular monitoring. The treatment of SLE mainly consists of antimalarial agents, glucocorticoids, non-biological immunosuppressants, and, more recently, biological agents, including B-cell-targeting therapies and a monoclonal antibody against the type I interferon receptor [5]. The recent approvals of new targeted therapies for SLE and lupus nephritis (LN), one of the most severe clinical manifestations of SLE [6], and the increasing awareness of the long-term adverse effects of glucocorticoids changed the focus of research towards the optimisation of therapeutic decision making, surveillance, and treatment evaluations, and technological advances paved the way for a cellular and molecular characterisation of SLE to serve as a basis for disease management [7,8]. In this context, identifying reliable biomarkers is imperative [9], and significant progress has been made in this area over the past few decades [10,11,12].

Historically, biomarker studies in SLE have focused on serum biomarkers [9,10,13,14]. Nevertheless, urinary, cerebrospinal fluid, and tissue biomarkers for organ-specific monitoring and prognostication are gaining increasing interest [12,15]. In this Special Issue titled “Immune Mechanisms and Biomarkers in Systemic Lupus Erythematosus”, we welcomed original works and review articles focusing on the cellular and molecular mechanisms underlying SLE in its different phases, with the goal of contributing novel knowledge of the pathogenesis of SLE and improved diagnostics, surveillance, prevention of long-term damage, and overall patient management. We also welcomed original works or review articles that evaluated immune components that could serve as diagnostic biomarkers, biomarkers of disease activity, or biomarkers of long-term outcomes. We received several contributions, and we hereby summarise the final content of the Special Issue, which is also visually represented in Figure 1.

In a cross-sectional study by Irene Carrión-Barberà et al., levels of advanced glycation end-products (AGEs) were found to be significantly higher in SLE patients compared to healthy controls. AGEs showed positive associations with the degree of SLE activity using the SLE disease activity index (SLEDAI) and the degree of organ damage using the Systemic Lupus International Collaborating Clinics (SLICC)/American College of Rheumatology (ACR) damage index (SDI), as well as various clinical features, indicating some potential of AGEs as biomarkers of monitoring and prognosis in SLE [16]. In a study by Bethany Wolf et al., significant differences in urine glycosphingolipids and N-glycans were observed between LN patients and healthy controls, with men showing more pronounced differences [17].

In a cohort study by Agnieszka Winikajtis-Burzyńska et al., elevated serum levels of soluble transferrin receptor (sTfR) were linked to an increased risk of cardiovascular, pulmonary, and haematological manifestations of SLE, while elevated interleukin (IL)-4 levels were associated with a decreased risk of mucocutaneous manifestations, collectively suggesting that sTfR and IL-4 could be useful for differentiating the risk of affliction across organ systems in SLE [18]. In an observational study of 284 SLE patients, Julia Mercader-Salvans et al. found that circulating levels of IL-6 were associated with a higher cardiovascular risk and disruption of the complement system, but not with SLE disease activity or organ damage [19]. Contrary to some prior observations [20,21], a study by Lina Wirestam et al. found no strong association between anti-oxidised low-density lipoprotein (anti-oxLDL) antibodies and vascular affliction in SLE patients [22]. Although a significant correlation was observed with intima media thickness in the common femoral artery, the overall findings do not support anti-oxLDL antibodies as reliable biomarkers for vascular involvement in SLE.

Using data from multiple phase III clinical trials of belimumab, Ioannis Parodis et al. investigated the role of early alterations in circulating B cell and plasma cell subsets in relation to renal flares in SLE patients treated for active extra-renal disease with non-biological standard therapy plus belimumab or placebo. A rapid decrease in short-lived plasma cells or plasmablasts with a subsequent return was associated with renal flares. Rapid decreases in transitional B cells and long-lived plasma cells upon belimumab therapy indicated greater protection against renal flares, which was not the case for the placebo-treated group of patients [23]. Collectively, B cell and plasma cell kinetics during the early phases of treatment with belimumab for active SLE might be useful early indicators of the need for therapeutic adjustments [24].

In a review by Matthieu Halfon et al., mitochondrial dysfunction was detailed as a significant factor in SLE pathogenesis, particularly in LN. Altered mitochondrial homeostasis and defective mitophagy appears to contribute to immune dysregulation, suggesting mitochondria as potential biomarkers and therapeutic targets in SLE and LN [25]. Based on the current literature, Alessandra Maria Vitale et al. proposed that molecular mimicry between human heat shock proteins (HSPs) belonging to the chaperone system and proteins and metabolites of gut commensal bacteria could link gut microbiota dysbiosis with SLE pathogenesis. The production of autoantibodies against HSPs, which are known to associate with SLE onset and progression, due to shared epitopes between human HSPs and those of gut commensal bacteria, may contribute to disease pathogenesis, warranting a coordinated study of these factors in SLE [26].

In a comprehensive review by Susannah von Hofsten et al., the roles of Toll-like receptors (TLRs) in SLE pathogenesis were discussed, particularly TLR7, TLR8, and TLR9. In brief, the authors detailed the link between the overexpression of TLR7 and SLE severity and further stated that TLR8 and TLR9 may regulate TLR7 activity, offering insights into potential therapeutic strategies targeting these receptors [27]. Last but not least, Fatima K. Alduraibi and George C. Tsokos provided a critical evaluation of biomarkers for LN. Despite improvements in kidney and patient survival, complete clinical and histological remission rates remain limited in LN, highlighting the need for the timely detection of kidney affliction due to SLE and the prompt initiation of therapy, as well as understanding of the specific attributes of proposed LN biomarkers, to further improve patient outcomes and guide disease management [28]. Importantly, despite advancements in biomarker research, kidney biopsy remains the gold standard for the determination of LN activity and the identification of histological features that dictate the pharmacotherapeutic need and guide management.

In summary, our Special Issue provides valuable insights into immune mechanisms and biomarkers relevant to SLE and LN, highlighting recent advancements in understanding the pathogenesis and improving diagnostics and patient management. The collected original works and reviews offer a comprehensive view of the potential biomarkers and therapeutic targets, paving the way for enhanced surveillance, the prevention of long-term organ damage, and optimised therapeutic decision making in SLE.

## Figures and Tables

**Figure 1:**
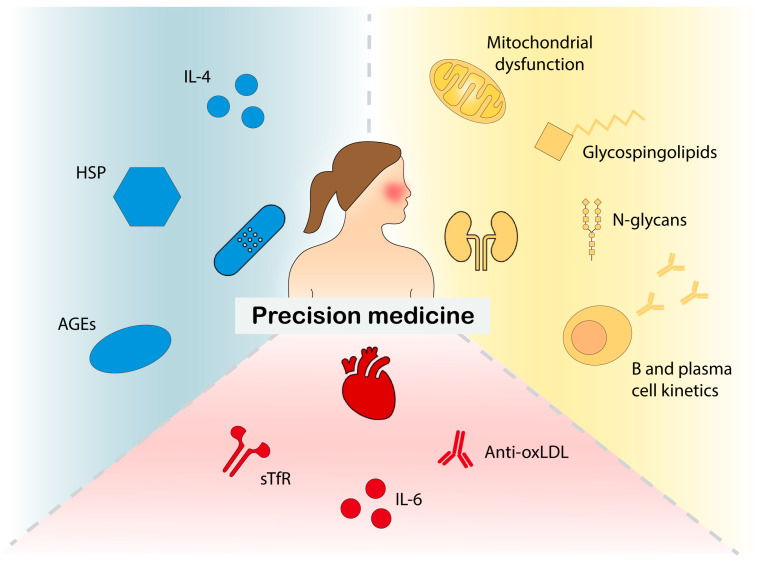
Visual representation of the content of the Special Issue. AGEs: advanced glycation end-products; anti-oxLDL: anti-oxidised low-density lipoprotein; HSP: heat shock protein; IL: interleukin; sTfR: soluble transferrin.
